# Intravitreal aflibercept 8 mg in patients from Japan with neovascular age-related macular degeneration: 48-week subgroup analysis of the PULSAR trial

**DOI:** 10.1007/s10384-025-01270-8

**Published:** 2025-12-26

**Authors:** Hideki Koizumi, Shigeru Honda, Tsutomu Yasukawa, Genichiro Kishino, Tetsuju Sekiryu, Andrea Schulze, Takuto Yamashita, Ursula Schmidt-Ott, Min Zhao, Xin Zhang, Alyson J. Berliner, Karen W. Chu, Kimberly Reed, Yenchieh Cheng, Rafia Bhore, Robert Vitti, Ikuko Fujita, Sergio Leal, Tomohiro Iida

**Affiliations:** 1 Department of Ophthalmology, Graduate School of Medicine, University of the Ryukyus, Okinawa, Japan; 2 Department of Ophthalmology and Visual Sciences, Graduate School of Medicine, Osaka Metropolitan University, Osaka, Japan; 3 Department of Ophthalmology and Visual Science, Nagoya City University Graduate School of Medical Sciences, Nagoya, Japan; 4 Kozawa Eye Hospital and Diabetes Center, Ibaraki, Japan; 5 Department of Ophthalmology, Fukushima Medical University, Fukushima, Japan; 6 Bayer AG, Berlin, Germany; 7 Bayer Yakuhin, Ltd., Osaka, Japan; 8 Bayer Healthcare Co. Ltd., Beijing, China; 9 Bayer Consumer Care AG, Basel, Switzerland; 10 Regeneron Pharmaceuticals, Inc., Tarrytown, NY USA; 11 Department of Ophthalmology, Tokyo Women’s Medical University, Shinjuku-ku, Tokyo, Japan; 12 Department of Ophthalmology, Ageo Central General Hospital, Saitama, Japan

**Keywords:** Aflibercept, Neovascular age-related macular degeneration, Anti-VEGF agent, Vascular endothelial growth factor

## Abstract

**Purpose:**

To evaluate the 1-year efficacy and safety of aflibercept 8 mg compared with aflibercept 2 mg in a pre-specified analysis of patients from Japan with neovascular age-related macular degeneration (nAMD) included in PULSAR.

**Study design:**

PULSAR (NCT04423718) was a global, phase 3, randomized, double-masked, non-inferiority study of adults with nAMD. Patients were randomized 1:1:1 to receive aflibercept 8 mg every 12 weeks (8q12), or every 16 weeks (8q16), or aflibercept 2 mg every 8 weeks (2q8), following three initial monthly doses in all groups.

**Methods:**

This subgroup analysis of Japan and non-Japan cohorts from PULSAR evaluated changes from baseline in best-corrected visual acuity (BCVA), central subfield retinal thickness, durability and safety outcomes.

**Results:**

In the Japan subgroup, least squares (LS) mean (95% CI) changes from baseline in BCVA at week 48 were +6.5 (+0.7, +12.3), +7.9 (+5.1, +10.6), and +4.7 (–0.5, +9.9) letters for patients in the 8q12 (n = 31), 8q16 (n = 33), and 2q8 (n = 33) groups, respectively. The majority of patients in the 8q12 (82.1%) and 8q16 (93.8%) groups maintained their randomized dosing intervals through Week 48. Ocular treatment-emergent adverse events were reported in 35.5%, 30.3%, and 39.4% of patients in the Japan subgroup in 8q12, 8q16, and 2q8 groups, respectively. Similar efficacy and safety results were observed in the non-Japan subgroup.

**Conclusion:**

Aflibercept 8 mg has similar efficacy and safety to aflibercept 2 mg when administered at extended dosing intervals in both the Japan and non-Japan subgroups, consistent with the overall PULSAR results.

**Supplementary Information:**

The online version contains supplementary material available at 10.1007/s10384-025-01270-8.

## Introduction

Age-related macular degeneration (AMD) is a leading cause of visual impairment globally [1]. Increasing age is a consistent risk factor for the development and progression of AMD; as such, AMD is a significant public health concern in countries with ageing populations, such as Japan, where almost 30% of the population are aged ≥65 years [2]. In Japan, the prevalence of AMD in older persons ranges from 13 to 22% [3], and is expected to increase based on projections of aging populations in Asia to over 100 million patients by 2040 [4]. The phenotypic features of neovascular AMD (nAMD) are reported to differ between Asian and Caucasian patients, with pachychoroid, polypoidal choroidal vasculopathy (PCV), and type 1 macular neovascularization being reported as more prevalent in Asian patients [5–8].

Currently approved treatments in Japan for nAMD include intravitreal injections of agents targeting the vascular endothelial growth factor (VEGF) signaling pathway (such as aflibercept, ranibizumab, brolucizumab, and faricimab), which are generally administered as required (pro re nata) or in a treat-and-extend paradigm [9]. The burden associated with treatment (including amongst many factors, waiting time during appointments, and out-of-pocket costs), has a considerable impact on patients, families and caregivers, and physicians and healthcare systems [10]. Thus, there is a significant unmet need to prevent or slow disease progression, and to reduce the burden on patients and clinics. Treatments that allow for extended dosing intervals would be of benefit in helping to alleviate burden to the patient (with respect to treatment and appointment attendance), and to the clinic (thereby improving capacity to allow flexibility in treating more patients) [10].

The efficacy and safety of aflibercept 2 mg has previously been demonstrated in patients with nAMD in Japan in the phase 3 VIEW 2 pivotal trial and the open-label phase 4 ALTAIR trial [11, 12]. An 8 mg formulation of aflibercept enabling the intravitreal delivery of a 4-times higher molar dose compared with the original 2 mg formulation has been developed to address the need to reduce the treatment burden while maintaining visual benefits. In the pivotal PULSAR trial in patients with nAMD, aflibercept 8 mg administered every 12 and 16 weeks (8q12 and 8q16) demonstrated non-inferiority in best-corrected visual acuity (BCVA) gains with a 4-letter margin at week 48 to aflibercept 2 mg administered every 8 weeks (2q8) [13]. By week 16, significantly more patients receiving aflibercept 8 mg were fluid-free (no intraretinal or subretinal fluid) in the center subfield vs. patients in the 2q8 group; fluid control in both groups was maintained through week 48 [13]. Approximately 80% of patients randomized to receive aflibercept 8 mg maintained their randomized 12-week and 16-week dosing intervals through week 48, and the rates of treatment-emergent adverse events were similar across treatment groups. Accordingly, aflibercept 8 mg has been approved for the treatment of nAMD by various regulatory agencies, including the US Food and Drug Administration [14], European Medicines Agency [15], and more recently (January 2024) by the Japanese Ministry of Health, Labour, and Welfare [16].

Here, we describe the results of a PULSAR subgroup analysis evaluating efficacy, durability, and safety of aflibercept 8 mg in patients from Japan, which will be of value in understanding the specific effects of aflibercept 8 mg in the Japanese population.

## Methods

### Study design

PULSAR (NCT04423718) was a randomized, phase 3, double-masked, non-inferiority, 3-arm, 96-week clinical trial of adults treated with aflibercept for nAMD. The full study details and 48-week results have been previously published [13]. PULSAR was conducted in 223 clinical sites across 27 countries, in accordance with the Declaration of Helsinki, International Council for Harmonization Good Clinical Practice Guidelines, and any applicable local laws and regulations. The study protocol was approved by relevant institutional review boards and independent ethics committees prior to initiation of the study (Online Resource 1).

Adults aged ≥50 years were eligible; one eye per patient was designated as the study eye [13]. Eligible patients were randomized into either aflibercept 2q8, 8q12, or 8q16 treatment groups in a 1:1:1 ratio, and commenced treatment with three initial monthly doses.

Beginning at week 16, patients receiving aflibercept 8 mg were assessed by the investigator at dosing visits according to the following dose regimen modification (DRM) criteria denoting disease activity: > 5-letter loss in BCVA from week 12, and either > 25-µm increase in central retinal thickness (CRT), or new foveal hemorrhage or neovascularization. Patients in the aflibercept 8q12 or 8q16 groups who had met the prespecified DRM criteria at week 16 or week 20 had their dosing interval shortened to receive aflibercept 8 mg every 8 weeks. Patients in the aflibercept 8q16 group who met DRM criteria at or after week 24 had their dosing intervals shortened by a 4-week increment. Patients randomized to the 2q8 group could not have their dosing intervals shortened. The minimum treatment interval for all patients was 8 weeks. Dosing intervals could not be extended in the first year of treatment. Patients were monitored every 4 weeks.

### Outcomes

This prespecified analysis evaluated functional, anatomic, and safety outcomes in two subgroups: patients enrolled from Japan (Japan subgroup) and patients enrolled from outside of Japan (non-Japan subgroup). Efficacy endpoints included changes from baseline in BCVA (Early Treatment Diabetic Retinopathy Score [ETDRS] letter score) at week 48, proportion of patients with no fluid (defined as no intraretinal [IRF] and no subretinal [SRF] fluid in the center subfield) at week 16, changes from baseline in CRT, measured from the inner limiting membrane to the retinal pigment epithelium) from baseline to week 48. The durability endpoint included the proportion of patients in the 8q12 or 8q16 groups who maintained their 12- or 16-week dosing intervals through week 48, respectively. Safety endpoints included the incidence of ocular and non-ocular treatment-emergent adverse events (TEAEs) and serious TEAEs through week 48.

### Statistical analysis

All efficacy analyses were performed in the full analysis set (FAS), which included all patients randomized  to treatment (participants were analyzed within their original randomized group) and who received ≥1 dose of study treatment. Safety variables were analyzed in the safety analysis set (SAF), which included all patients randomly assigned and who received ≥1 dose of study treatment (participants were analyzed as treated). No statistical comparisons were performed between the Japan and non-Japan subgroups, nor between aflibercept 8 mg or aflibercept 2 mg within these subgroups.

The primary efficacy endpoint analysis in these subgroups was conducted using the same methodology used in the overall PULSAR population [13]. Data points after the first occurrence of relevant intercurrent events were excluded from this analysis; missing data were handled using a mixed model for repeated measures (MMRM). Differences in least squares (LS) mean change from baseline in BCVA at week 48 between the 8q12 and 2q8 groups and between the 8q16 and 2q8 groups were calculated using an MMRM that included baseline BCVA as covariate and treatment group and stratification variables (baseline BCVA [<60 vs. ≥60 letters]) as fixed factors, as well as terms for the interaction between baseline BCVA and visit and for the interaction between treatment and visit. A similar analysis was conducted to evaluate changes in CRT using baseline CRT (instead of baseline BCVA) as a covariate, and terms for the interaction between baseline CRT and visit (instead of baseline BCVA and visit).

The last observation carried forward (LOCF) method was used to impute missing values for patients who had at least one post-baseline value and had any further missing post-baseline data points until week 48 for analysis of categorical variables (proportion of patients without retinal fluid in the center subfield at week 16, week 48). The proportion of patients who maintained their treatment interval, and the number of injections per treatment group, were analyzed descriptively based on the subset of patients who completed the week 48 visit. Safety endpoints were summarized descriptively. Statistical evaluation was performed using the software package SAS version 9.4 or higher.

## Results

### Patients

In the PULSAR trial, a total of 1009 patients received treatment. Of these, 97 patients had been enrolled in Japan (Japan subgroup) and were included in the FAS and SAF (8q12: n = 31; 8q16 n = 33; 2q8: n = 33) for this analysis (Table 1). In total, 91 (93.8%) patients in the Japan subgroup completed treatment through week 48 (Supplementary Figure 1). Out of 1009 patients, 912 patients enrolled in PULSAR from outside of Japan (non-Japan subgroup) received treatment (8q12 n = 304, 8q16 n = 305, and 2q8: n = 303) and were included in the FAS and SAF (Supplementary Fig. 1). In total, 846 patients (92.8%) in the non-Japan subgroup completed treatment through week 48.

**Table 1:** Baseline demographics and disease characteristics (study eye) of the Japan and non-Japan subgroup in the PULSAR trial

	Japan (n = 97)				Non-Japan (n = 912)			
	Aflibercept 2q8 (n = 33)	Aflibercept 8q12 (n = 31)	Aflibercept 8q16 (n = 33)	Combined aflibercept 8 mg (n = 64)	Aflibercept 2q8 (n = 303)	Aflibercept 8q12 (n = 304)	Aflibercept 8q16 (n = 305)	Combined aflibercept 8 mg (n = 609)
Age, years	71.2 (9.4)	73.8 (7.4)	74.7 (7.8)	74.3 (7.6)	74.5 (8.7)	74.8 (8.0)	74.4 (8.6)	74.6 (8.3)
Sex, n (%)								
Female	9 (27.3)	8 (25.8)	5 (15.2)	13 (20.3)	179 (59.1)	174 (57.2)	175 (57.4)	349 (57.3)
Male	24 (72.7)	23 (74.2)	28 (84.8)	51 (79.7)	124 (40.9)	130 (42.8)	130 (42.6)	260 (42.7)
Race, n (%)								
Asian	33 (100)	31 (100)	33 (100)	64 (100)	50 (6.5)	43 (14.1)	44 (14.4)	87 (14.3)
Black Or African American	0	0	0	0	2 (0.7)	2 (0.7)	0	2 (0.3)
Multiple	0	0	0	0	0	1 (0.3)	0	1 (0.2)
Not Reported	0	0	0	0	2 (0.7)	2 (0.7)	1 (0.3)	3 (0.5)
White	0	0	0	0	249 (82.2)	256 (84.2)	260 (85.2)	516 (84.7)
BCVA, ETDRS letter score	60.6 (14.6)	61.5 (12.8)	59.5 (12.7)	60.5 (12.7)	58.8 (14.0)	59.7 (13.4)	60.1 (12.4)	59.9 (12.9)
CRT, μm	320 (112)	349 (112)	342 (87)	345 (99)	372 (135)	373 (125)	374 (136)	373 (131)
CNV size, mm^2^	5.3 (4.0)	6.1 (5.6)	5.6 (5.1)	5.8 (5.3)	6.5 (5.1)	6.0 (4.8)	6.7 (5.6)	6.3 (5.2)
Type of CNV, n (%)								
Predominantly classic	4 (12.1)	3 (9.7)	3 (9.1)	6 (9.4)	67 (22.1)	68 (22.4)	64 (21.0)	132 (21.7)
Minimally classic	6 (18.2)	7 (22.6)	5 (15.2)	12 (18.8)	55 (18.2)	49 (16.1)	63 (20.7)	112 (18.4)
Occult only	21 (63.6)	19 (61.3)	21 (63.6)	40 (62.5)	171 (56.4)	178 (58.6)	165 (54.1)	343 (56.3)
PCV presence (ICGA confirmed), n/N (%)^a^	15/23 (65.2)	13/22 (59.1)	14/25 (56.0)	27/47 (57.4)	39/84 (46.4)	31/76 (40.8)	27/62 (43.5)	58/138 (42.0)

Full analysis set. ^a^ICGA was not performed for all patients; as such, the actual percentages of PCV may be higher than reported here. Continuous data are summarized by mean (SD) and categorical data by frequency counts and percentages. CRT was measured from the inner limiting membrane to the retinal pigment epithelium. CNV reported as per reading center. In Japan, 98 patients were randomized and 97 were treated. 2q8=aflibercept 2 mg every 8 weeks. 8q12=aflibercept 8 mg every 12 weeks. 8q16=aflibercept 8 mg every 16 weeks. *BCVA* best-corrected visual acuity; *CNV* choroidal neovascularization; *CRT* central retinal thickness; *ETDRS* Early Treatment Diabetic Retinopathy Study; ICGA, indocyanine green angiography; *N* number of patients with ICGA assessment; *PCV* polypoidal choroidal vasculopathy

Baseline demographics and disease characteristics, including BCVA and CRT, were consistent across treatment groups within the Japan subgroup as well as the non-Japan subgroup (Table 1**).** There were comparatively fewer women and a lower proportion of predominantly classic choroidal neovascularization (CNV) in the Japan subgroup compared with the non-Japan subgroup. In the Japan subgroup, PCV was confirmed in 59.1%, 56.0% and 65.2% of patients in the 8q12, 8q16, and 2q8 groups who underwent indocyanine green angiography (ICGA), respectively. Among patients in the non-Japan subgroup who underwent ICGA, PCV was confirmed in 40.8%, 43.5%, and 46.4% of patients in the 8q12, 8q16, and 2q8 groups, respectively.

Patients in the 8q12, and 8q16, and 2q8 groups in the Japan subgroup who completed the week 48 visit received a mean (SD) of 6.1 (0.3), 5.0 (0) and 7.0 (0) aflibercept injections, respectively. Patients in the non-Japan subgroup, in the 8q12, 8q16, and 2q8 groups who completed the week 48 visit received a mean (SD) of 6.1 (0.4), and 5.2 (0.7), and 6.9 (0.3) aflibercept injections, respectively.

### Functional outcomes

**Fig. 1:**
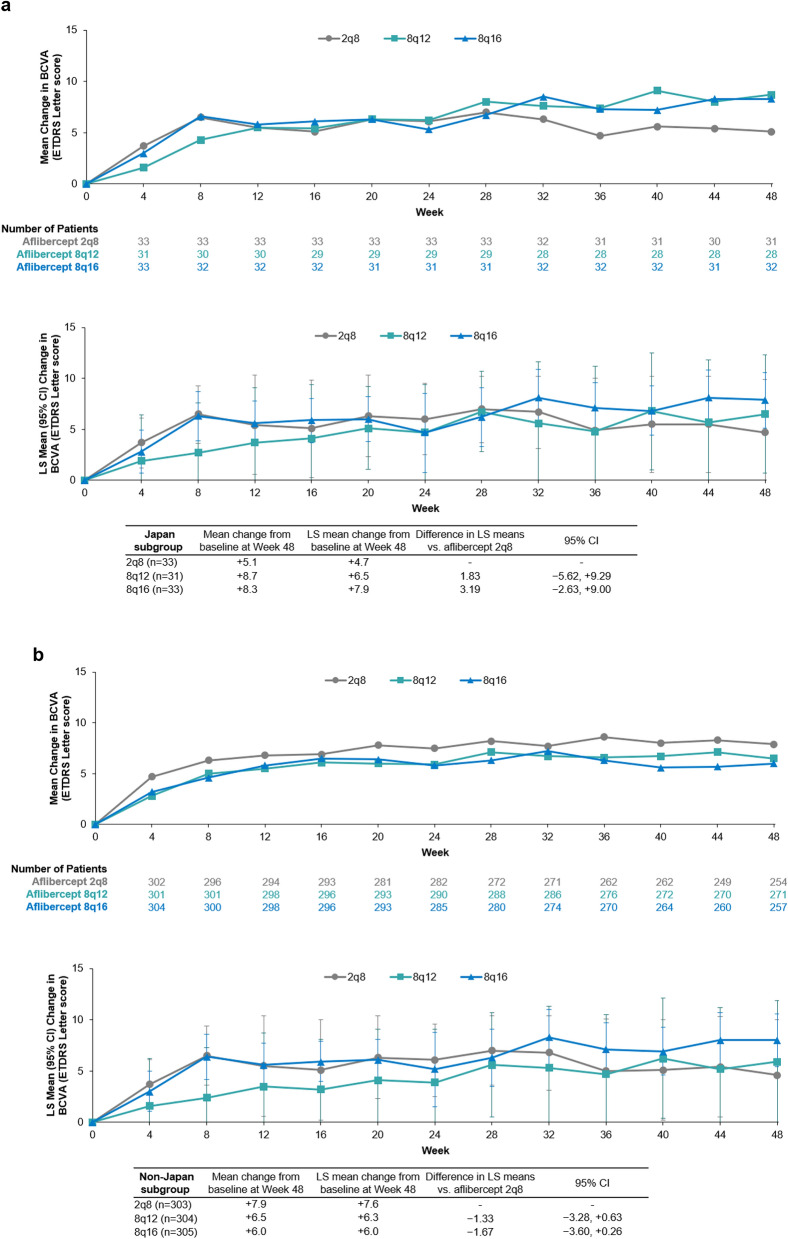
Arithmetic mean change and LS mean (95% CI) change in BCVA by visit through 48 weeks of aflibercept treatment in the (**a)** Japan and (**b)** non-Japan subgroups. Full analysis set. Arithmetic mean changes based on OC prior to relevant ICE, LS mean changes based on MMRM. 2q8=aflibercept 2 mg every 8 weeks. 8q12=aflibercept 8 mg every 12 weeks. 8q16=aflibercept 8 mg every 16 weeks. *BCVA* best-corrected visual acuity. CI confidence interval. *ETDRS* Early Treatment Diabetic Retinopathy Study. *ICE* intercurrent event. *LS* least squares. *MMRM* mixed model repeated measures. *OC* observed cases.

An improvement in BCVA was observed in all three treatment groups (aflibercept 8q12, 8q16, and 2q8) in the Japan and non-Japan subgroups through 48 weeks of aflibercept treatment (Fig. 1). In the Japan subgroup, mean (SD) change from baseline in BCVA at week 48 was +8.7 (12.2), +8.3 (7.4), and +5.1 (14.9) letters for patients in the aflibercept 8q12, 8q16, and 2q8 groups, respectively (Fig.1a). In the Japan subgroup, LS mean (95% CI) change from baseline was +6.5 (+0.7, +12.3), +7.9 (+5.1, +10.6), and +4.7 (–0.5, +9.9) letters for patients in the aflibercept 8q12, 8q16, and 2q8 groups, respectively. The difference in LS means (95% CI) was +1.8 (–5.6, +9.3) between 8q12 and 2q8, and +3.2 (–2.6, +9.0) between 8q16 and 2q8.

In the non-Japan subgroup, mean (SD) change from baseline in BCVA at week 48 was +6.5 (12.6), +6.0 (12.1), +7.9 (11.8) letters for patients in the aflibercept 8q12, 8q16, and 2q8 groups, respectively (Fig. 1b). LS mean (95% CI) change from baseline was +6.3 (+4.9, +7.7), +6.0 (+4.6, +7.4), and +7.6 (+6.3, +9.0) for patients in the aflibercept 8q12, 8q16, and 2q8 groups, respectively, from the non-Japan subgroup. The difference in LS means (95% CI) was –1.3 (–3.3, +0.6) between 8q12 and 2q8, and –1.7 (–3.6, +0.3) between 8q16 and 2q8.

### Anatomic outcomes

**Fig. 2:**
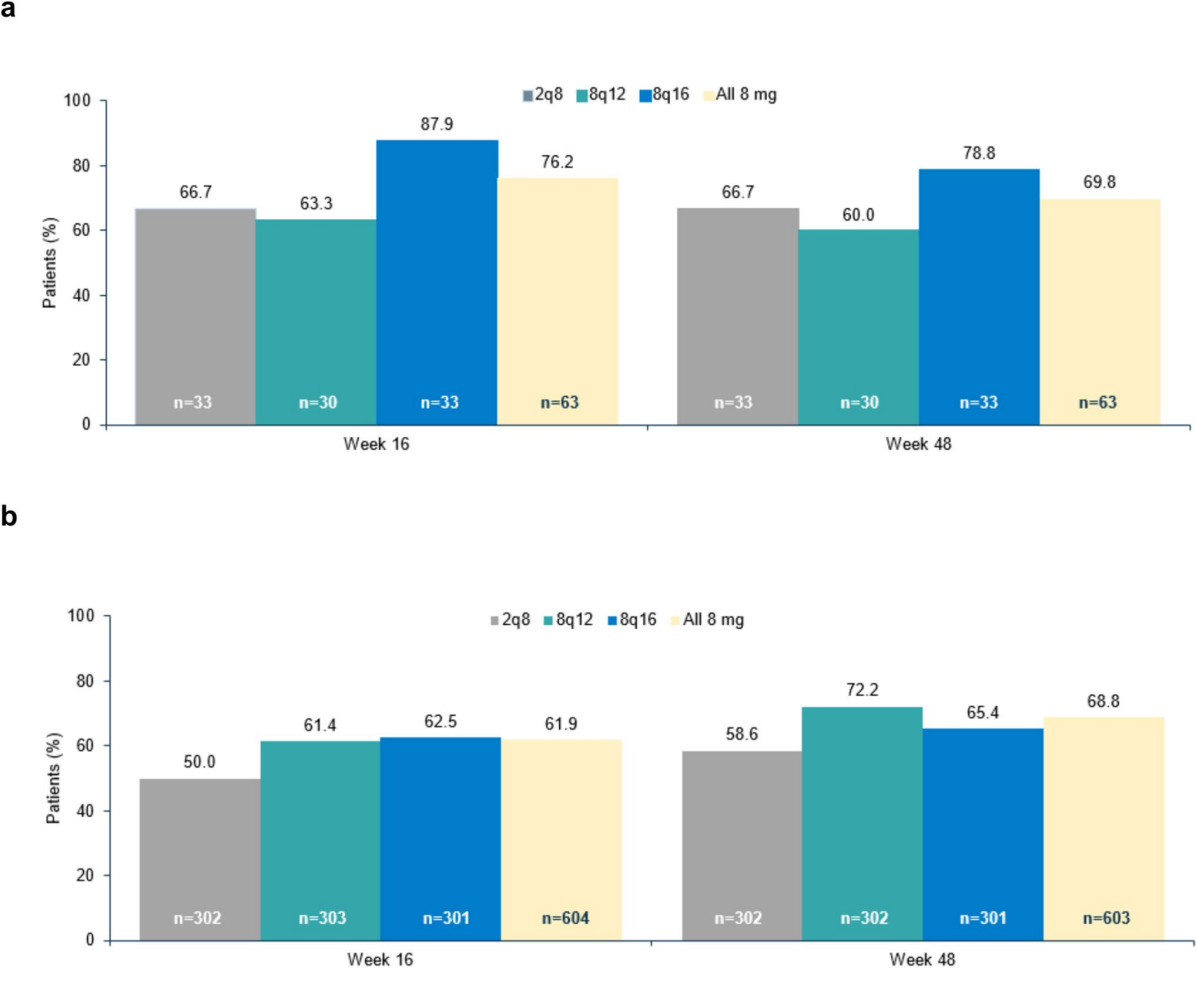
Proportion of patients without retinal fluid in the central subfield at week 16 and week 48 in the (**a)** Japan and (**b)** non-Japan subgroups. Full analysis set. Last observation carried forwards. One patient in the 8q12 cohort was missing/ungradable at weeks 16 and 48. 2q8=aflibercept 2 mg every 8 weeks. 8q12=aflibercept 8 mg every 12 weeks. 8q16=aflibercept 8 mg every 16 weeks.

In the Japan subgroup, 76.2% of patients receiving aflibercept 8 mg had no retinal fluid (subretinal or intraretinal) present in the central subfield at week 16 (8 weeks following three initial monthly injections in each treatment group), compared with 66.7% in the aflibercept 2q8 group (Fig. 2a). In the non-Japan subgroup, 61.9% of patients receiving aflibercept 8 mg had no retinal fluid in the central subfield at week 16, compared with 50.0% in the 2q8 group (Fig. 2b). Fluid outcomes at week 16 was sustained at week 48 in all treatment groups in both the Japan and non-Japan subgroups (Fig. 2a and b).

**Fig. 3:**
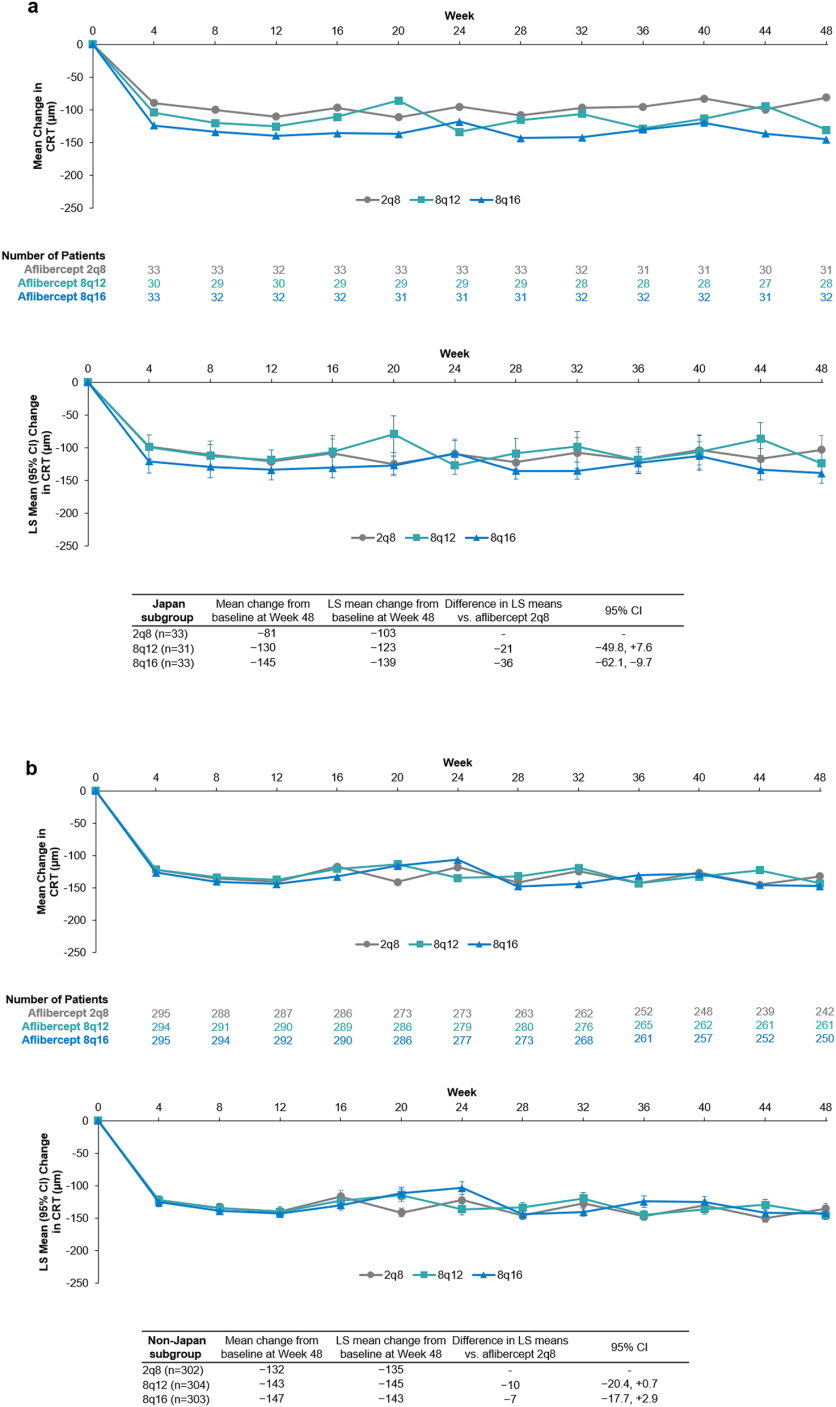
Arithmetic mean change and LS mean change (95% CI) in CRT by visit over 48 weeks of aflibercept treatment in the (**a**) Japan and (**b)** non-Japan subgroups. Full analysis set. Arithmetic mean changes based on OC prior to relevant ICE, LS mean changes based on MMRM. 2q8=aflibercept 2 mg every 8 weeks. 8q12=aflibercept 8 mg every 12 weeks. 8q16=aflibercept 8 mg every 16 weeks. *CI* confidence interval. *CRT* central retinal thickness. *ICE* intercurrent event. *LS* least squares. *MMRM* mixed model repeated measures. *OC* observed cases

Reductions in mean CRT were observed in all three treatment groups in the Japan subgroup following 48 weeks of aflibercept treatment **(**Fig. 3a**)**. Mean (SD) change from baseline in CRT (µm) at week 48 was –130 (114), –145 (93), and –81 (112) for patients in the 8q12, 8q16, and 2q8 groups, respectively. The LS mean (95% CI) changes from baseline in CRT (µm) to week 48 were ‒124 (–143, –104), –139 (–154, –123), and –103 (–125, –81) for those patients in the 8q12, 8q16, and 2q8 groups, respectively.

In the non-Japan subgroup, mean (SD) change from baseline in CRT (µm) at week 48 was –143﻿ (121), –147 (136), and –132 (125) for patients who were in the 8q12, 8q16, and 2q8 groups, respectively. LS mean changes from baseline in CRT (µm) to week 48 were –145 (–152, –138), –143 (–149, –136), and –135 (–143, –127) for patients in the 8q12, 8q16, and 2q8 groups, respectively.

### Durability

In the Japan and non-Japan subgroups, respectively, 92% and 82% of patients who received aflibercept 8 mg maintained ≥12-week dosing intervals through week 48, following the initial monthly injections.

**Fig. 4:**
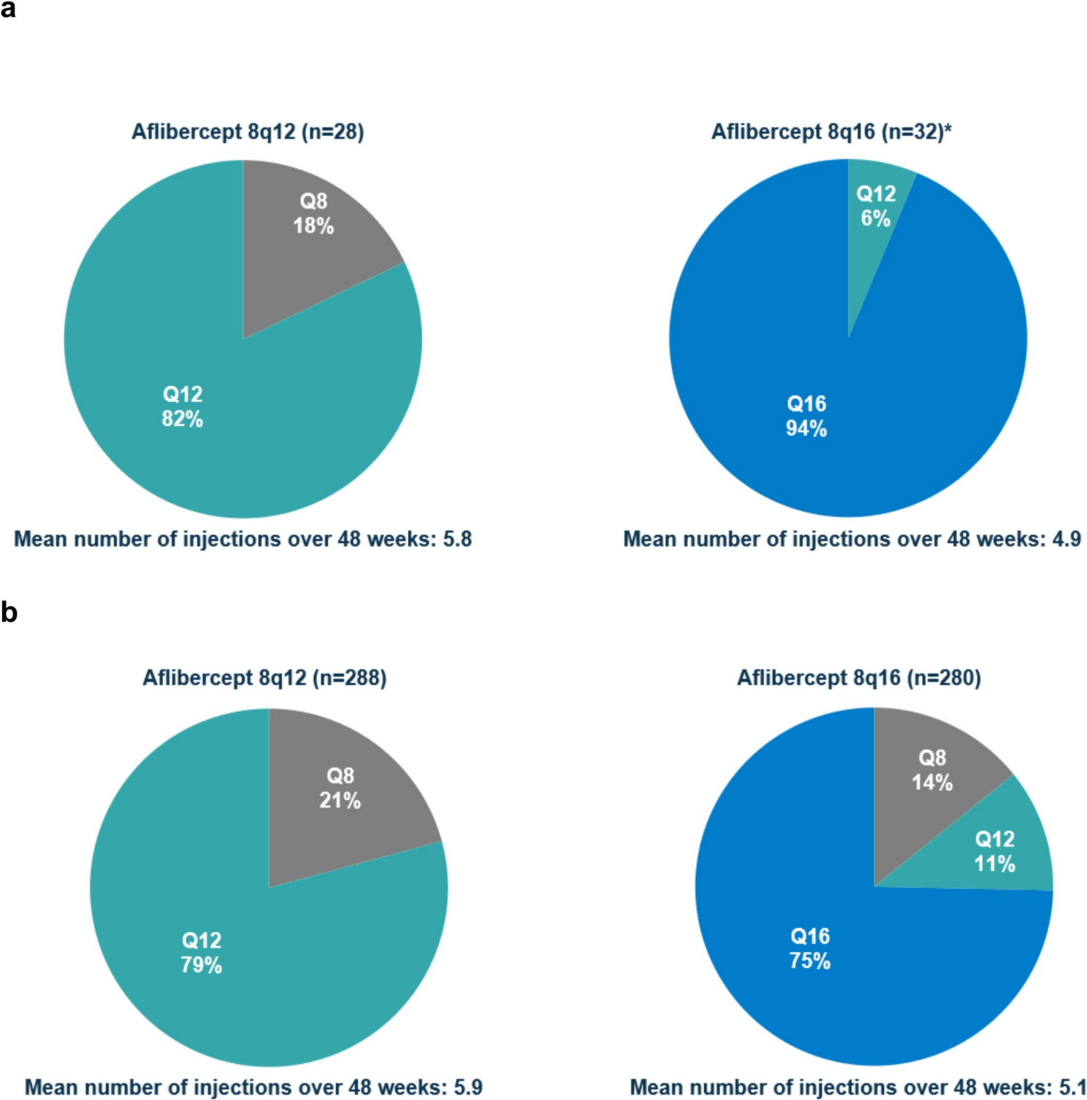
Proportion of patients in the (**a)** Japan and (**b)** non-Japan subgroups receiving aflibercept 8 mg who maintained their randomized dosing intervals through week 48. *No patients in the 8q16 group from the Japan subgroup required their dosing intervals to be shortened to Q8. Data presented for patients who completed 48 weeks of treatment and completed the week 48 visit of the trial. 2q8=aflibercept 2 mg every 8 weeks. 8q12=aflibercept 8 mg every 12 weeks. 8q16=aflibercept 8 mg every 16 weeks. Q4, every 4 weeks. Q8, every 8 weeks. Q12, every 12 weeks. Q16, every 16 weeks.

In the Japan subgroup, 82% of patients in the 8q12 group maintained their original assigned 12-week dosing interval through week 48. In the Japan 8q16 group, 94% of patients maintained their original assigned 16-week dosing interval, and 100% maintained ≥12-week dosing intervals through week 48 (Fig. 4a). In the non-Japan subgroup, 79% of patients in the 8q12 group maintained their original assigned 12-week dosing interval through week 48. In the non-Japan 8q16 group 75% of patients maintained their original assigned 16-week dosing interval, and 86% maintained ≥12-week dosing intervals through week 48 (Fig. 4b).

### Safety

The safety profiles of aflibercept 8 mg and 2 mg were comparable in the Japan and non-Japan subgroups (Table 2). In the Japan subgroup, the proportion of patients with ocular TEAEs was comparable in patients receiving 8 mg and those in the 2q8 group (8q12: 35.5%; 8q16: 30.3%; and 2q8: 39.4%) (Table 2). Ocular TEAEs occurring in ≥5% of patients in any treatment arm were conjunctival hemorrhage, dry eye, intraocular pressure increased, retinal hemorrhage, seasonal allergy, and visual acuity reduced (Supplementary Table 1). In the Japan sub-group, serious ocular TEAEs were reported in 1 patient in the 8q16 group (angle closure glaucoma), and 1 patient in the 2q8 group (retinal hemorrhage); none were reported in the 8q12 group.

**Table 2:** Summary of key adverse events up to week 48 of PULSAR in Japan and non-Japan subgroups

	Japan (n=97)				Non-Japan (n=912)			
	Aflibercept 2q8 (n=33)	Aflibercept 8q12 (n=31)	Aflibercept 8q16 (n=33)	Combined aflibercept 8 mg (n=64)	Aflibercept 2q8 (n=303)	Aflibercept 8q12 (n=304)	Aflibercept 8q16 (n=305)	Combined aflibercept 8 mg (n=609)
Ocular TEAE in the study eye, n (%)								
Any ocular TEAE	13 (39.4)	11 (35.5)	10 (30.3)	21 (32.8)	117 (38.6)	118 (38.8)	117 (38.4)	235 (38.6)
Any ocular serious TEAE	1 (3.0)	0	1 (3.0)	1 (1.6)	1 (0.3)	6 (2.0)	4 (1.3)	10 (1.6)
Non-ocular TEAE, n (%)								
Any non-ocular TEAE	18 (54.5)	23 (74.2)	19 (57.6)	42 (65.6)	160 (52.8)	152 (50.0)	163 (53.4)	315 (51.7)
Any non-ocular serious TEAE	3 (9.1)	4 (12.9)	4 (12.1)	8 (12.5)	43 (14.2)	30 (9.9)	28 (9.2)	58 (9.5)
Any TEAE of intraocular inflammation in the study eye, n (%)								
Iridocyclitis	0	0	0	0	1 (0.3)	0	1 (0.3)	1 (0.2)
Vitreal cells	0	0	0	0	1 (0.3)	1 (0.3)	0	1 (0.2)
Chorioretinitis	0	0	0	0	0	1 (0.3)	0	1 (0.2)
Iritis	0	1 (3.2)	0	1 (1.6)	0	0	0	0
Vitritis	0	0	0	0	0	1 (0.3)	0	1 (0.2)
Intraocular pressure increase	1 (3.0)	0	4 (12.1)	4 (6.3)	6 (2.0)	11 (3.6)	5 (1.6)	16 (2.6)

Safety analysis set. *TEAE* treatment-emergent adverse event. One patient (0.3%) in each treatment group in the non-Japan subgroup was uncoded for ocular TEAEs of the study eye

In the non-Japan subgroup, the proportion of patients with TEAEs was also comparable across subgroups (8q12: 38.8%; 8q16: 38.4%; and 2q8: 38.6%). Ocular TEAEs occurring in ≥5% of patients in any treatment arm were visual acuity reduced (Supplementary Table 1). In the non-Japan subgroup, serious ocular TEAEs were reported in 6 patients in the 8q12 group (1 report each of cataract, choroidal detachment, retinal hemorrhage; 2 reports of increased intraocular pressure; and 3 reports of retinal detachment), 4 patients in the 8q16 group (1 report each of retinal detachment, retinal hemorrhage, skin laceration, and vitreous hemorrhage), and 1 patient in the 2q8 group (1 report of angle closure glaucoma).

One case of intraocular inflammation (iritis) was reported in a patient from Japan through week 48 (Table 2). This occurred in the 8q12 group, and was considered by the study investigator to be mild in severity, not serious, and unrelated to the study drug. The iritis resolved prior to week 48, and did not lead to treatment discontinuation. No cases of endophthalmitis, occlusive retinal vasculitis, vitritis, chorioretinitis, or iridocyclitis were reported in the Japan subgroup. In the non-Japan subgroup, single cases of vitreal cells, chorioretinitis, and vitritis were reported in the 8q12 group, iridocyclitis in the 8q16 group, and iridocyclitis and vitreal cells in the 2q8 group. In the Japan subgroup, intraocular pressure (IOP) increased (AE as reported by investigators) was reported in numerically more patients in the 8q16 group (4/33 [12.1%]) compared with the 2q8 group (1/33 [3.0%]) and the 8q12 group (0/31 [0%]) (Table 2); these cases were considered mild in severity by the study investigator. In the non-Japan subgroup, increased intraocular pressure was reported in 11/304 (3.6%) of patients from the 8q12 group, 5/305 (1.6%) of patients from the 8q16 group, and 6/303 (2.0%) of patients from the 2q8 group (Table 2).

In the Japan subgroup, non-ocular TEAEs were observed in 74.2% of patients on 8q12, 57.6% of patients in the 8q16 group, and 54.5% of patients in the 2q8 group. No non-ocular TEAEs were reported in more than 5% of any group. Serious non-ocular TEAEs in the Japan subgroup occurred in 4/31 (12.9%) patients in the 8q12 group, 4/33 (12.1%) of patients in the 8q16 group, and 3/33 (9.1%) of patients in the 2q8 group and in 12.5% (8/64) of patients who received aflibercept 8 mg (Table 2). In the non-Japan subgroup, non-ocular TEAEs were observed in 50.0% of patients on 8q12, 53.4% of patients on 8q16, and 52.8% of patients on 2q8. Apart from COVID-19 infection (10/304 [3.3%] of patients in the 8q12 group, 21/305 [6.9%] of patients in the 8q16 group, and 11/303 [3.6%] of patients in the 2q8 group), no other non-ocular TEAEs were reported in ≥5% of patients in the non-Japan subgroup. In the non-Japan subgroup, non-ocular serious TEAEs occurred in 30/304 (9.9%) of patients in the 8q12 subgroup, 28/305 (9.2%) of patients in the 8q16 group, and 43/303 (14.2%) of patients in the 2q8 subgroup.

Overall, 9 deaths were reported in the PULSAR study through week 48; one death was reported for a patient from the Japan subgroup in the 2q8 group, and 8 deaths for non-Japan patients randomized to the 8q12 (n = 3) and 8q16 (n = 1), and 2q8 (n = 4) treatment groups. None of the deaths was considered related to aflibercept.

## Discussion

This subgroup analysis of PULSAR evaluated the efficacy, durability, and safety of aflibercept 8 mg in patients from Japan.

Robust gains in BCVA (ranging from +4.7 to +7.9 letters) were observed at week 48 in patients in the Japan subgroup treated with aflibercept 8 mg and aflibercept 2 mg). Similar robust improvements in BCVA were also observed in the non-Japan subgroup in patients receiving aflibercept 8 mg and aflibercept 2 mg (ranging from +6.0 to +7.9 letters), consistent with the overall PULSAR population [13]. Comparable reductions in CRT were observed across the 8 mg and 2 mg groups in the Japan (ranging from –103 to –139 µm) and non-Japan (ranging from –135 to –145 µm) subgroups at week 48, and rapid fluid resolution was apparent at week 16 and was sustained at week 48. Of note, the comparable functional and anatomic outcomes across the treatment groups were achieved with fewer injections of aflibercept 8 mg compared with aflibercept 2 mg in both the Japan and non-Japan subgroups, consistent with findings in the overall PULSAR population. Approximately 92% of patients who were randomized to receive aflibercept 8 mg and completed week 48 in the Japan subgroup maintained dosing intervals of 12 weeks or longer through week 48, demonstrating the durability of aflibercept 8 mg in being able to adequately control disease with extended dosing intervals in this patient population.

Safety findings indicate that the profile of aflibercept 8 mg in the Japan subgroup was comparable to that of aflibercept 2 mg, consistent with results from the non-Japan subgroup and the overall PULSAR population [13]. A single case of intraocular inflammation, and no cases of endophthalmitis or occlusive retinal vasculitis, were reported in the Japan subgroup. As no increased IOP was reported in the Japan 8q12 group, where injections of a similar volume were given more frequently than in the 8q16 group, the increase in the number of patients with increased IOP in the 8q16 group was considered to be a random finding and not driven by the drug, the injection procedure, or higher injection volume (compared with aflibercept 2 mg). Such an imbalance in IOP events between the 8q12 and 8q16 groups was not observed in the overall study population.

Limitations of this study include those inherent to any subgroup analysis of a study (for example, inadequate power for comparisons), relatively small sample size of participants from Japan, the one-year follow-up, the lack of adjustment for multiplicity and that due to the asynchronous dosing across the aflibercept 8q12, 8q16, and 2q8 groups after week 8, direct comparison of treatment effects is not feasible. In addition, variables such as sub-retinal pigment epithelium fluid were not assessed in the study. Whilst the data in this study provide important insights into the response of patients from Japan to aflibercept 8 mg, a follow-up study at week 96 will be important in describing long-term outcomes in this subpopulation.

The results of this subgroup analysis indicate that treatment with aflibercept 8 mg administered at extended dosing intervals was comparable to aflibercept 2 mg in terms of improvements in visual acuity, anatomic outcomes and fluid resolution in both the Japan and non-Japan patients, with no new safety concerns. These results are consistent with the overall PULSAR population.

## Supplementary Information

Below is the link to the electronic supplementary material.

Supplementary file2 (DOCX 16 KB)

Supplementary file2 (DOCX 504 KB)
