# Supplementary material for: Intravitreal aflibercept 8 mg in patients from Japan with neovascular age-related macular degeneration: 48-week subgroup analysis of the PULSAR trial
Source: Jpn J Ophthalmol. 2025 Dec 26;70(1):139–49. doi: 10.1007/s10384-025-01270-8 (PMC12948824; doi:10.1007/s10384-025-01270-8)
Supplement: Supplementary file 1 — Supplementary file2 (DOCX 16 KB) [file 10384_2025_1270_MOESM1_ESM.docx]

**Online Resource 1. List of institutional review boards and ethics committees.**

Comité Independiente de Ética , Argentina; St Vincent's Hospital (Melbourne) Human Research Ethics Committee, Australia; Medizinische Universität Graz, Ethik-Kommission, Austria; Ethics Committee for Clinical Trials, Bulgaria; Western University Research Ethic Committee, Canada; The University Of Manitoba Biomedical Research Ethics Board, Canada; Shanghai General Hospital Institutional Review Board, China; Medical Ethics Committee of Union Hospital affiliated to Tongji Medical College of Huazhong University of Science and Technology, China; Medical Ethics Committee of Henan Provincial Ophthalmology Hospital, China; Ethics Committee of The First Affiliated Hospital of Xinjiang Medical University, China; Ethical Committee for Clinical Trial Review of Tianjin Eye Hospital, China; Beijing Hospital Ethics Committee, China; The Medical Ethics Committee of The Second Hospital of Jilin University, China; Drug Ethical Committee of Tianjin Medical University General Hospital, China; Drug Clinical Trial Ethics Committee of Huazhong University of Science and Technology, China; Ethics Committee of Eye Hospital Affiliated to Wenzhou Medical University, China; Clinical Trial Ethics Committee of The People's Hospital of Ningxia Hui Autonomous Region, China; Ethics Committee of Guangzhou Aier Eye Hospital, China; Medical Ethics Committee of The First Affiliated Hospital of Nanchang University, China; Ethics Committee of Beijing Aier Intech Eye Hospital Co., Ltd, China; Chengdu Aier Eye Hospital Drug Clinical Trial Ethics Committee, China; Medical Ethics Committee of Jiangxi Provincial People's Hospital, China; The Medical Ethics Committee of Shenyang He Eye Hospital, China; Ethics Committee of Biomedical Study of Peking University First Hospital, China; Medical Ethics Committee of Zhejiang Provincial People's Hospital, China; Ethics Review Committee for Registration Oriented Clinical Trials of the First Affiliated Hospital of Zhejiang University School of Medicine, China; Clinical Trial Branch of Medical Ethics Committee of the First Affiliated Hospital of Fujian Medical University, China; The Clinical Trials Ethics Committee of Shenyang Aire Eye Optometry Hospital, China; Medical Ethics Review Committee of Chongqing Aier Ophthalmology Hospital, China; Ethics Committee of Capital Medical University (CMU) - Beijing Tongren Hospital, China; Ethics Review Committee of Peking University People's Hospital, China; Medical Ethics Committee of Tianjin Medical University Eye Hospital, China; Medical Ethics Committee of First Affiliated Hospital of Xi'an Jiaotong University, China; Drug Clinical Trial Ethics Committee of The Second Hospital of Anhui Medical University, China; Medical Ethics Committee of Xuzhou First People's Hospital, China; Clinical Trial Ethics Committee of Hebei Eye Hospital, China; Institutional Review Board of Shanghai Tongji Hospital, China; Drug Clinical Trial Ethics Committee of Tonghua Central Hospital, China; Medical Ethics Committee of Shijiazhuang People's Hospital, China; Eticka komise Axon Clinical, Czech Republic; Fakultni nemocnice Ostrava, Czech Republic; FN Hradec Kralove, Czech Republic; FN Kralovske Vinohrady, Czech Republic; Lexum a.s., Czech Republic; Fakultni nemocnice Plzen, Czech Republic; Fakultni nemocnice Brno, Czech Republic; Ethics committee for medicinal products, Estonia; CPP SUD-Ouest et Outre-Mer II, France; EC of , Inova “LTD-6 Z.Anjaparidze ST first lane, Georgia; EC of Chichua Medical Center „Mzera“, Georgia; EC of Caucasus Medical Centre, Georgia; EC of LTD -„Clinic LJ“, Georgia; Medical Research Council Ethics Committee for Clinical Pharmacology (ECCP), Hungary; LEC of Meir Medical Center, Israel; Helsinki Committee of The Chaim Sheba Medical Center, Israel; The Helsinki committee of Assuta Medical Center, Israel; Helsinki Committee of Shamir Medical Center (Assaf Harofeh), Israel; Helsinki Committee of Barzilai Medical Center, Israel; Helsinki Committee of The Lady Davis Carmel Medical Center, Israel; Helsinki Committee of Rambam Health Care Campus, Israel; Helsinki Committee of Kaplan Medical Center, Israel; Helsinki Committee of Hadassah Medical Organization, Israel; Comitato Etico Unico Regionale Piazza Ospedale, Italy; Comitato Etico IRCCS Ospedale San Raffaele, Italy; Comitato Etico Indipendente presso la Fondazione Policlinico Tor Vergata di Roma, Italy; Comitato Etico Centrale Irccs – Sezione I.F.O.– Fondazione Bietti, Italy; CER Umbria - Comitato Etico Regionale Umbriapresso CREO, Italy; Comitato Etico Catania 1, Italy; Comitato Etico Indipendente AOU di Cagliari, Italy; Comitato Etico della Fondazione Policlinico Universitario Agostino Gemelli IRCCS Università Cattolica del Sacro Cuore, Italy; Comitato Etico Milano Area 1 – Asst Sacco Fatebenefratelli, Italy; Kobe University Hospital – IRB, Japan; Hyogo Prefectural Amagasaki General Medical Center IRB, Japan; Hayashi Eye Hospital IRB, Japan; Kokura Memorial Hospital – IRB, Japan; Nagoya City University Hospital IRB, Japan; ACTIVATO Institutional Review Board, Japan; Nara Medical University Hospital IRB, Japan; IRB of Okayama University Hospital, Japan; University of the Ryukyus Hospital IRB, Japan; Joint IRB, Japan; IRB of Shiga University of Medical Science Hospital, Japan; Mie University hospital IRB, Japan; Akita University Hospital IRB, Japan; Review Board of Human Rights and Ethics for Clinical Studies Institutional Review Board, Japan; Nagoya University Hospital IRB, Japan; Kansai Medical University Hospital IRB, Japan; Hiroshima University Hospital IRB, Japan; Yamaguchi University Hospital IRB, Japan; Aomori Prefectural Central Hospital IRB, Japan; National Hospital Organization National Tokyo Medical Center IRB, Japan; ShinAkasaka Clinic Institutional Review Board, Japan; Kanazawa University Hospital IRB, Japan; ShinAkasaka Clinic Aoyama Institutional Review Board, Japan; Tokushima University Hospital IRB, Japan; Oita University Hospital IRB, Japan; Kozawa Eye Hospital and Diabetes Center Institutional Review Board, Japan; Institutional Review Board of Tohoku Medical and Pharmaceutical University Hospital, Japan; The University of Tokyo Hospital IRB, Japan; University of Yamanashi Hospital IRB, Japan; Juntendo University Urayasu Hospital, Japan; Shinshu University Hospital IRB, Japan; Kansai Medical University MC IRB, Japan; Japanese Red Cross Nagasaki Genbaku Hospital IRB, Japan; Kanazawa Medical University Hospital, Japan; Jimbo Orthopedics IRB, Japan; Osaka Metropolitan Univ Hospital IRB, Japan; Sumitomo Hospital – IRB, Japan; Kindai University Hospital – IRB, Japan; Aichi Medical University Hospital IRB, Japan; Japanese Red Cross Saitama Hospital Institutional Review Board, Japan; University of Miyazaki Hospital IRB, Japan; St. Luke’s International Hospital IRB, Japan; Keio University Hospital IRB, Japan; ShinAkasaka Clinic Aoyama Institutional Review Board, Japan; Wakayama MC – IRB, Japan; Kagawa University Hospital IRB, Japan; Kurashiki Medical Center IRB, Japan; Gunma University Hospital Institutional Review Board, Japan; Southern Tohoku General Hospital IRB, Japan; Tokyo Women's Medical University Hospital IRB, Japan; Yamagata University Hospital IRB, Japan; Jimbo Orthopedics IRB, Japan; Sapporo City General Hospital IRB, Japan; St. Marianna University Group IRB, Japan; Tokyo Medical University Hachiouji Medical Center IRB, Japan; KKR Sapporo Medical Center IRB, Japan; Pusan National University Hospital, South Korea; Asan Medical Center, South Korea; Samsung Med Ctr IRB #1, South Korea; Seoul National U Hosp IRB #1, South Korea; Seoul National University Bund, South Korea; Korea University Guro Hospital, South Korea; Inje University Haeundae Paik, South Korea; Nune Eye Hospital, South Korea; Ethics Committee for Clinical Recearch at Pauls Stradins Clinical University Hospital Development Society, Latvia; EC, Studentu 45A, Lithuania; CEIC - Comissão de Ética para a Investigação Clínica Avª Do Brasil, Portugal; Scientific Research Institution, Russia; Fgu Intersectoral Research And Technology Complex, Russia; Samara State Medical Universit, Russia; Irkutsk Branch of FBSI IRTC E, Russia; Ethics Committee Of Serbia, Serbia; SingHealth Centralized IRB #1, Singapore; Fakultna nemocnica Trencin, Slovakia; Univerzitna nemocnica Bratislava, Slovakia; Fakultna nemocnica s poliklinik, Slovakia; Fakultna nemocnica Nitra, Slovakia; NsP Trebisov a.s., Slovakia; CEIm Hospital Universitari de Bellvitge, C/ Feixa Llarga, s/n - Antiguo Módulo Banco de Santander, Spain; Ethik-Kommission Nordwest- und Zentralschweiz, Switzerland; Chang Gung Medical Foundation - Linkou Chang Gung Memorial H, Taiwan; Shin Kong Wu Ho-Su Memorial Hospital - IRB/IEC, Taiwan; Kaohsiung Medical University - Chung-Ho Memorial Hospital, Taiwan; Taipei Veterans General Hospital, Taiwan; Changhua Christian Hospital, Taiwan; Institute of Eye Diseases and Tissue Therapy named after V.P, Ukraine; Poltava Central Regional Clinical Hospital, Ukraine; Centralna Polyclinica MVS Ukraiiny, Ukraine; Likuvalno-diahnostychnyi tsentr pryvatnoho pidpryiemstva pry, Ukraine; Advarra, United States of America; Vanderbilt (Human Research Protection Program HRPP), United States of America; The University of Vermont Committees on Human Subjects, United States of America; Wake Forest School of Medicine, United States of America; Kaiser Permanente Southern California, United States of America.
