# Supplementary material for: Intravitreal aflibercept 8 mg in patients from Japan with neovascular age-related macular degeneration: 48-week subgroup analysis of the PULSAR trial
Source: Jpn J Ophthalmol. 2025 Dec 26;70(1):139–49. doi: 10.1007/s10384-025-01270-8 (PMC12948824; doi:10.1007/s10384-025-01270-8)
Supplement: Supplementary file 2 — Supplementary file2 (DOCX 504 KB) [file 10384_2025_1270_MOESM2_ESM.docx]

**Supplementary Table 1:** Ocular TEAEs occurring in >5% of any treatment up to week 48 of PULSAR in the Japan and non-Japan subgroups

|  | **Japan (n=97)** | | | | **Non-Japan (n=912)** | | | |
| --- | --- | --- | --- | --- | --- | --- | --- | --- |
|  | **Aflibercept**  **2q8 (n=33)** | **Aflibercept 8q12 (n=31)** | **Aflibercept 8q16 (n=33)** | **Combined aflibercept  8 mg  (n=64)** | **Aflibercept  2q8**  **(n=303)** | **Aflibercept 8q12**  **(n=304)** | **Aflibercept 8q16**  **(n=305)** | **Combined aflibercept 8 mg**  **(n=609)** |
| Ocular TEAEs occurring in >5% of any treatment group, n (%) |  |  |  |  |  |  |  |  |
| Conjunctival hemorrhage | 1 (3.0) | 3 (9.7) | 1 (3.0) | 4 (6.3) | 4 (1.3) | 5 (1.6) | 4 (1.3) | 9 (1.5) |
| Dry eye | 2 (6.1) | 0 | 1 (3.0) | 1 (1.6) | 2 (0.7) | 5 (1.6) | 4 (1.3) | 9 (1.5) |
| Retinal hemorrhage | 2 (6.1) | 1 (3.2) | 0 | 1 (1.6) | 12 (4.0) | 10 (3.3) | 10 (3.3) | 20 (3.3) |
| Visual acuity reduced | 4 (12.1) | 2 (6.5) | 1 (3.0) | 3 (4.7) | 16 (5.3) | 10 (3.3) | 17 (5.6) | 27 (4.4) |
| Seasonal allergy | 2 (6.1) | 0 | 0 | 0 | 0 | 0 | 0 | 0 |

Safety analysis set. TEAE, treatment-emergent adverse event. One patient (0.3%) in each treatment group of non-Japan patients was uncoded for ocular TEAEs of the study eye.

**Supplementary Figure 1:** Trial profile of the Japan and non-Japan subgroups of PULSAR.


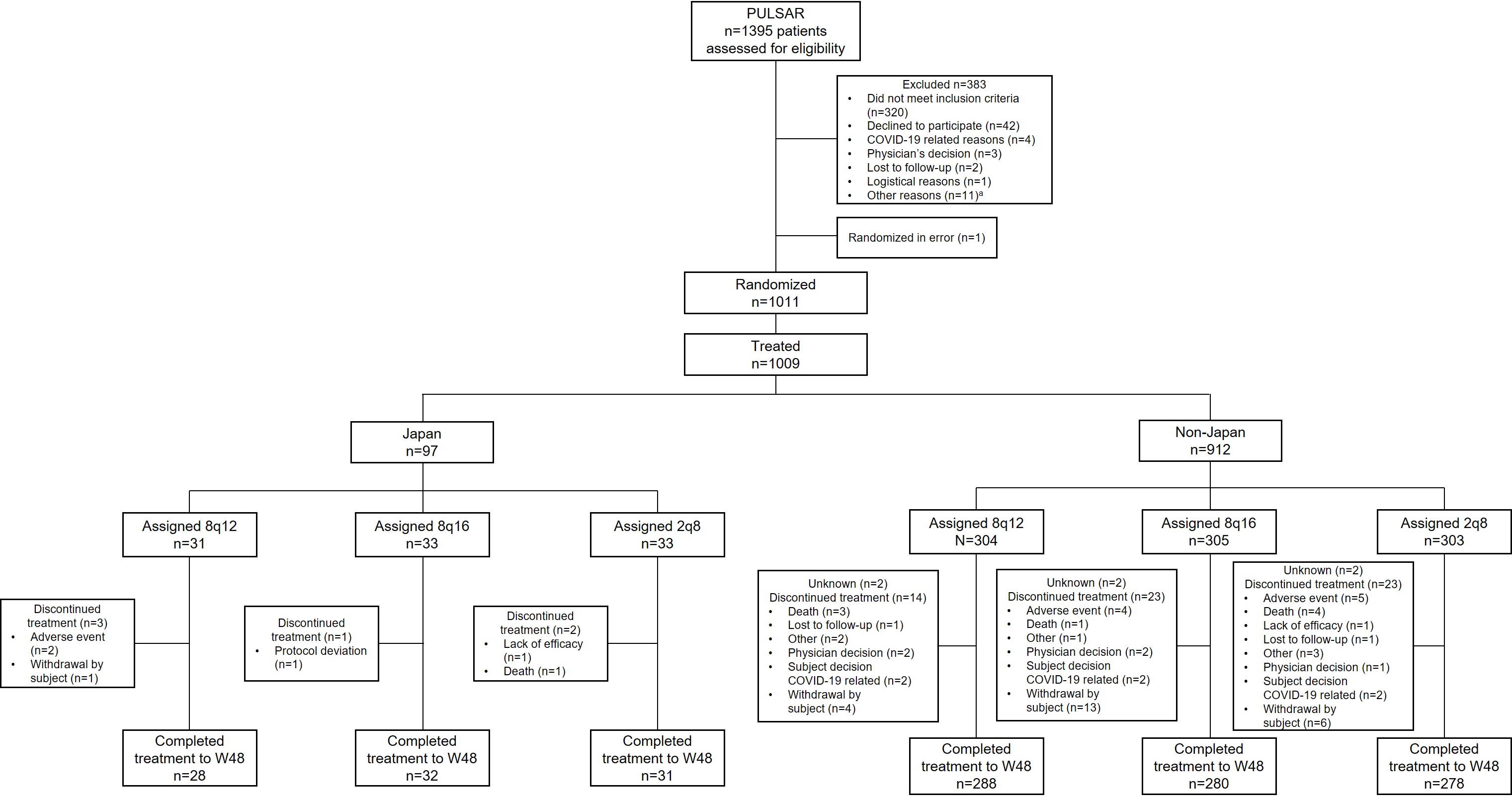


^a^Please refer to Lanzetta et al., *Lancet* 2024 for more information on patients excluded [13]. 2q8, aflibercept 2 mg every 8 weeks; 8q12, aflibercept 8 mg every 12 weeks; 8q16, aflibercept 8 mg every 16 weeks; COVID-19, coronavirus 2019; W, week.
